# Revisiting a pollen-transmitted ilarvirus previously associated with angular mosaic of grapevine

**DOI:** 10.1016/j.virusres.2024.199362

**Published:** 2024-03-22

**Authors:** Mathieu Mahillon, Justine Brodard, Ruben Schoen, Marleen Botermans, Nathalie Dubuis, Raphaël Groux, John R. Pannell, Arnaud G. Blouin, Olivier Schumpp

**Affiliations:** a Research group Virology, Bacteriology and Phytoplasmology, Department of Plant protection, Agroscope, Nyon, Switzerland; b Netherlands Institute for Vectors, Invasive plants and Plant health (NIVIP), Netherlands Food and Consumer Product Safety Authority, Wageningen, The Netherlands; c Department of Ecology and Evolution, University of Lausanne (UNIL), Switzerland

**Keywords:** *Bromoviridae*, Grapevine, *Ilarvirus*, *Mercurialis*, Pollen, *Orthotospovirus*, Thrips

## Abstract

We report the characterization of a novel tri-segmented RNA virus infecting *Mercurialis annua*, a common crop weed and model species in plant science. The virus, named “Mercurialis latent virus” (MeLaV) was first identified in a mixed infection with the recently described Mercurialis orthotospovirus 1 (MerV1) on symptomatic plants grown in glasshouses in Lausanne (Switzerland). Both viruses were found to be transmitted by *Thrips tabaci*, which presumably help the inoculation of infected pollen in the case of MeLaV. Complete genome sequencing of the latter revealed a typical ilarviral architecture and close phylogenetic relationship with members of the *Ilarvirus* subgroup 1. Surprisingly, a short portion of MeLaV replicase was found to be identical to the partial sequence of grapevine angular mosaic virus (GAMV) reported in Greece in the early 1990s. However, we have compiled data that challenge the involvement of GAMV in angular mosaic of grapevine, and we propose alternative causal agents for this disorder. In parallel, three highly-conserved MeLaV isolates were identified in symptomatic leaf samples in The Netherlands, including a herbarium sample collected in 1991. The virus was also traced in diverse RNA sequencing datasets from 2013 to 2020, corresponding to transcriptomic analyses of *M. annua* and other plant species from five European countries, as well as metaviromics analyses of bees in Belgium. Additional hosts are thus expected for MeLaV, yet we argue that infected pollen grains have likely contaminated several sequencing datasets and may have caused the initial characterization of MeLaV as GAMV.

## Highlights

• MeLaV is a new ilarvirus of the subgroup 1 infecting the weed *Mercurialis annua*.
• The virus was found with MerV1 on diseased glasshouse-grown plants in Switzerland.
• *Thrips tabaci* is a vector of MerV1 and contributes to the pollen transmission of MeLaV.
• MeLaV may have been mistakenly associated with a grapevine disorder in Greece.
• The virus was traced in plant and bee samples across Europe, as far back as 1991.

## 1 Introduction

Plant viruses pose substantial threats to global food and feed production as they are frequently associated with the emergence and resurgence of diseases (Rojas and Gilbertson, 2008). Most of these viruses circulate within agroecosystems using vector organisms, which allow them to alternate between crop and non-crop plants (Bragard et al., 2013; Alcalá-Briseño et al., 2020; Rivarez et al., 2023a; Schönegger et al., 2023). Thus, in order to mitigate the impact of viral pathologies, it is imperative to undertake viromics surveys encompassing economically important species, but also wild plants that may serve as reservoirs (Ma et al., 2020; McLeish et al., 2022). Specifically, weeds that surround and invade crop fields represent valuable targets for virological studies (Chao et al., 2022). Herein, we conducted a comprehensive characterization of a novel ilarvirus infecting the ubiquitous weed *Mercurialis annua*.

The herbaceous *M. annua*, commonly referred to as “annual mercury” is a species from the family *Euphorbiaceae* originally found in Europe and around the Mediterranean Basin (Pannell et al., 2008). It is a toxic ruderal that has been linked to cases of poisoning in cattle and pollen allergy in humans (Welchman et al., 1995; Vallverdú et al., 1997). It is considered an agricultural weed because it can readily invade crop fields, thanks to high dispersal ability and seed persistence. The species occurs in a polyploid complex, with striking variation in its sexual systems. Diploid populations of the species complex, which are distributed from the eastern Mediterranean Basin across Europe to the North Sea, are dioecious, whereas hexaploid and tetraploid populations, which occur in the Iberian Peninsula and northwestern Africa, are monoecious or androdioecious (with males co-existing with hermaphrodites) (Durand, 1963; Obbard et al., 2006). The species has been used in studies of plant ecology, population genetics, and evolution, including the evolution of sex chromosomes (Pannell et al., 2004; Cossard et al., 2021; Gerchen et al., 2022). *M. annua* is not grown as a crop, hence disease reports have been limited. Nevertheless, the species is known to host damaging viral pathogens such as tomato spotted wilt virus, sowbane mosaic virus and tomato yellow leaf curl virus (Abou-Jawdah and Shebaro, 1999; Grieco et al., 2000; Atakan et al., 2013; Kazinczi et al., 2004). Recently, annual mercuries collected in tomato-associated agroecosystems in Slovenia were found to be infected with Mercurialis orthotospovirus 1 (MerV1), a tri-segmented ambisense RNA virus that belongs to the Iris yellow spot virus (IYSV) clade of the genus *Orthotospovirus* (Rivarez et al., 2023a). Thus, in addition to its harmful effect as weed, *M. annua* represents a potential source of known and novel viral pathogens, reinforcing the need for additional investigation on its virome.

The genus *Ilarvirus*, belonging to the family *Bromoviridae* in the order *Martellivirales*, constitutes a significant group of plant viruses known for their detrimental impacts (Bos et al., 1980; Podolyan et al., 2020). For instance, tobacco streak virus (TSV) is widely distributed and responsible for severe diseases on numerous hosts (Reddy et al., 2002; Sharman and Thomas, 2015). Another example is Parietaria mottle virus (PMV) which threatens solanaceous crops in the Mediterranean Basin (Parrella et al., 2016; Parrella et al., 2020). Several ilarviruses also impact the production of stone fruits and berries worldwide (Pallas et al., 2012; Martin et al., 2012; Tzanetakis and Martin, 2013; Noda et al., 2017). At the molecular level, these viruses exhibit a tri-segmented, positive sense, single-stranded (+ss) RNA genome (Bujarski et al., 2019). The longest RNA (RNA1, *c.* 3.4 kb) produces 1a, a protein with hypothetical methylase (MET) and helicase (HEL) activities (Pallas et al., 2013). The second RNA (RNA2, *c.* 3.1 kb) encodes 2a that is an RNA-dependent RNA polymerase (RdRp). An additional ORF producing an RNA silencing suppressor (RSS or 2b) is found on the RNA2 of several ilarviruses and is translated from a subgenomic RNA (sgRNA) (Xin et al., 1998; Shimura et al., 2013). The smallest RNA (RNA3, *c.* 2.1 kb) encodes a movement protein (MP or 3a) and a capsid protein (CP or 3b). The CP, translated from a sgRNA, is essential for “activation” of the genome, allows for *in planta* movement and forms the virions (Pallas et al., 2013; Jaspars, 1999; He et al., 2023). Viral particles range from quasi-spherical to bacilliform, and can be unstable in leaf extracts. The prefix “ilar“ actually originates from the initial characterization of these **i**sometric and **la**bile virions causing **r**ingspot symptoms. Transmission has been demonstrated by natural and artificial grafting as well as via seeds (Bos et al., 1980; Kaiser et al., 1991), but one of the main routes of dissemination consists of infected pollen transported by wind and insects (Sdoodee and Teakle, 1993; Klose et al., 1996; Aramburu et al., 2010; Bristow and Martin, 1999). Ilarviral particles have been detected on the surface as well as inside the pollen grains, so that the entry in plants occurs either via fertilization or mechanically during the feeding of thrips (Sdoodee and Teakle, 1993; Aparicio et al., 1999).

In recent years, the accumulation of an enormous amount of high-throughput sequencing (HTS) data from diverse environments has significantly expanded the known global virome. In parallel, transcriptome mining tools have become increasingly proficient at screening extensive HTS datasets for the presence of viral RdRp, thus facilitating the discovery of new RNA viruses and tracing of old ones. This is illustrated for instance by Solanum nigrum ilarvirus 1 (SnlV1), a pathogen of several solanaceous species that has also been detected in HTS datasets from non-solanaceous plants and even from non-plant organisms (Orfanidou et al., 2022; Rivarez et al., 2023b). This virus is seed-borne and transmissible by sap and grafting, and it is most likely disseminated via infected pollen too. In fact, SlnV1-infected pollen is believed to have contaminated many HTS procedures, thereby explaining the presence of viral contigs in unexpected samples such as human blood and phyllosphere-inhabiting organisms (Rivarez et al., 2023b).

In the present study, we initially focused on symptomatic annual mercuries grown at the University of Lausanne (UNIL, Switzerland). These plants were analyzed at Agroscope (Switzerland), where they were found to be infected by the novel ilarvirus in mixed-infection with MerV1. Following complete genome sequencing of both viruses, we investigated their respective vector and host range. It appeared that the new ilarvirus might have been previously associated with a disease of grapevine (*Vitis vinifera*) in Greece, known as angular mosaic (AM) and first documented in 1994 (Girgis et al., 2009). Fortunately, we were able to re-examine this disorder using plant material provided to Agroscope and to the Netherlands Institute for Vectors, Invasive plants and Plant health (NIVIP). Interestingly, HTS surveys conducted at NIVIP identified the new ilarvirus in symptomatic leaves of two other plant species. Last, traces of this virus were detected in several HTS datasets across Europe, and we discuss the biological significance of these results.

## 2 Materials and methods

### 2.1 Plant material

Symptomatic plants of *M. annua* grown in glasshouses at the UNIL were collected in the summer 2021 and placed in glasshouses at Agroscope (20–24 °C with 14/10 h artificial light). The plants were infested with thrips and thus maintained in homemade insect-proof cages. Every two to three months, healthy *M. annua* seedlings (grown from seeds provided by the UNIL) were added to the cages to replace dead plants.

Grapevines affected by AM have been kindly provided by Dr. S. M. Girgis and Dr. P. E. Kyriakopoulou (Grapevine Institute of Athens, Greece) to Agroscope and NIVIP. At Agroscope, three grapevine canes have been grafted onto the “SO4” rootstock (*Vitis berlandieri* × *Vitis riparia*). In addition, multiple budwoods from the AM-affected vines have been double-grafted between the rootstock “3309 Coudrec” (*Vitis riparia* x *Vitis rupestris*) and two red scion-indicator cultivars independently (Pinot noir and Gamay rouge de la Loire). At NIVIP, grapevine plants were not grafted but directly used for HTS analyses (see Section 2.4).

In addition to AM-affected grapevines, three symptomatic leaf samples from two other species were analyzed in the frame of phytosanitary surveys at NIVIP. The first sample (6,934,090) was a zucchini (*Cucurbita pepo*) leaf collected during a field inspection in Limburg (The Netherlands) in 2017. The second sample (33,558,588) was another zucchini leaf collected in a field in Almeria (Spain) in 2018. These two leaf samples were used for the sap inoculation of cucumber (*Cucumis sativus* cv. “Chinese Slangen”) and zucchini, respectively, according to an EPPO protocol (EPPO, 2022). Leaves from these inoculated indicator plants were then used for HTS analyses (see Section 2.4). The last sample (WAG0452486) corresponded to a leaf of curuba (*Passiflora tripartita* var. *mollissima*) collected in 1991 from an unknown location in The Netherlands and conserved in a historical herbarium collection. This sample was directly used for HTS.

### 2.2 Mechanical inoculation

For the initial mechanical inoculation procedure conducted at Agroscope, leaves from the collected *M. annua* plants were ground in a cold phosphate buffer using a mortar and a pestle as previously described (Mahillon et al., 2023), and the resulting sap was mechanically rubbed onto the leaves of *Nicotiana benthamiana, N. tabacum* cv. Xanthi*, N. occidentalis, Chenopodium quinoa* and *C. amaranticolor*. For the host range analyses, the viruses were first separated on distinct hosts for which the infection status was checked by RT-PCR. MerV1 was inoculated using a leaf extract from mono-infected *M. annua*, while infected leaves of mono-infected *C. amaranticolor* were used for the ilarvirus.

### 2.3 Transmission electron microscopy

Two distinct methods were followed for the observation of virions by transmission electron microscopy (TEM). For MerV1, a rapid leaf-dip protocol was sufficient. Briefly, leaf samples *c.* 5 mm in diameter were first ground in one drop of 0.1 % bovine serum albumin and one drop of 4 % phosphotungstic acid (pH 6.0). The resulting mix was pulverized onto Formvar/Carbon 400-Mesh copper grids (Agar scientific) using a custom made device. Grids were then observed using the Tecnai G2 Spirit electron microscope.

For the ilarvirus, a semi-purification of the particles was necessary prior TEM analyses. To this end, a protocol developed for Prunus necrotic ringspot virus (PNRSV) was followed with some modifications (Fulton, 1968). Briefly, 20 g of infected leaves of *C. amaranticolor* were first ground in 30 ml of cold extraction buffer (20 mM Na_2_HPO_4_, pH 8.0, 20 mM beta-mercaptoethanol, 20 mM diethyldithiocarbamate). After slow mixing on ice for 20 min, the mixture was clarified by centrifugation at 10,000 rpm for 10 min. The supernatant was collected and the pH was adjusted to 4.6 with drops of 100 mM citric acid. After a second centrifugation at 10,000 rpm for 10 min, the supernatant was collected and the pH was adjusted to 6.5 with drops of 200 mM Na_2_HPO_4_ (pH 8.0). Then, a final ultracentrifugation step (2 h at 30,000 rpm) was performed, and the resulting pellet was suspended in 1 mL of resuspension buffer (10 mM Na_2_HPO_4_, pH 7.0, 10 mM MgCl_2_). Particles in the resulting suspension were visualized by TEM as described above.

### 2.4 High-throughput sequencing

Six HTS analyses were performed for this study; two at Agroscope and four at NIVIP.

At Agroscope, the first HTS analysis was conducted on leaves of AM-affected grapevine canes which had been grafted onto the SO4 rootstock. Total RNA was first extracted using a CTAB protocol (see Section 2.6), and treated with DNase I (Qiagen) to remove plant DNA. The treated RNA was then sent to Fasteris (Switzerland) for Illumina HTS. After a ribodepletion step, a 150-bp cDNA library was built and sequenced on a Hiseq 4000 platform. A total of 5 Gb of reads were generated, quality trimmed with Trimmomatic (Bolger et al., 2014), and used for *de novo* assembly using Spades (Prjibelski et al., 2020) and Trinity (Grabherr et al., 2011). The resulting contigs (> 200 bp) were then loaded as queries for online BlastN and BlastX searches in NCBI databases. The second HTS analysis conducted at Agroscope was performed on samples from leaves of *N. benthamiana* plants which had been inoculated using a sap produced from the symptomatic *M. annua* plants. A cellulose-based protocol was first used to extract the viral dsRNA as previously described (Mahillon et al., 2021), which was followed by treatment with DNase I and S1 nuclease (Thermo Scientific). The resulting extract was then mixed with an unrelated viral dsRNA extract from *Malva sylvestris*, for which the HTS analysis has been recently documented (Mahillon et al., 2023).

For the HTS analyses performed at NIVIP, total RNA was extracted from the leaf samples as previously described (Botermans et al., 2013). RNA extracts were then sent to GenomeScan (The Netherlands) for Illumina HTS. Samples were first ribo-depleted, and a 150-bp cDNA library was then built. Libraries from zucchini and cucumber samples were sequenced on a NovaSeq 500 platform, while a NovaSeq 6000 platform was used for the samples from grapevine and curuba. Each run generated 2 Gb of paired-end reads, which were analyzed with CLC Genomics workbench v. 12.0.1 (Qiagen) by a custom workflow designed for the detection of *de novo* assembled viral contigs (Hammond et al., 2021). Consensus sequences were analyzed using megaBLAST and Diamond (Buchfink et al., 2015) in combination with locally downloaded versions of the NCBI nr databases. Results were visualized with Krona (Ondov et al., 2011).

### 2.5 Viral genome analyses

Complete sequencing was achieved for the two viruses identified on sap-inoculated *N. benthamiana* plants at Agroscope. While a near full-length genome was compiled for MerV1 after the HTS analysis, only partial contigs were assembled for the ilarvirus and the gaps were thus closed by bridging RT-PCR using specific primers. For both viruses, the viral 5′-termini were sequenced by nested-PCR using the SMARTer RACE 5′/3′ kit (Takara) in combination with primers listed in Table S1. For the sequencing of 3′-termini, viral sequences were first amplified as previously described (Mahillon et al., 2023), then cloned in the pGemT-easy vector (Promega) and sent to Fasteris (Switzerland) for Sanger sequencing using M13 universal primers.

Viral genomes were analyzed on UGene (Okonechnikov et al., 2012) and Jalview (Waterhouse et al., 2009). RNA secondary structures were predicted on the Mfold web server with default parameters (Zuker, 2003). For phylogenetic analyses, protein sequences were first retrieved from NCBI databases and aligned with Muscle (Edgar, 2004). Next, optimal substitution models for these alignments were determined with ModelFinder (Kalyaanamoorthy et al., 2017). Maximum-likelihood (ML) phylogenetic trees were then constructed via IQ-Tree (Nguyen et al., 2015) in combination with ultrafast bootstrap (Hoang et al., 2018). The resulting ML trees were manually curated on ITol (Letunic and Bork, 2019).

The NCBI Sequence Read Archive (SRA) database was screened for viral RdRp via the palmID tool of the Serratus cloud computing infrastructure (Edgar et al., 2022). A haplotype network was then constructed for an alignment of long (> 1 kb) ilarviral contigs using the R packages Ape and Pegas (Paradis, 2010; Paradis and Schliep, 2019).

### 2.6 Detection of DNA and RNA

For the analyses conducted at Agroscope, nucleic acids were extracted from plant and insect tissues using a 3 % CTAB protocol (Mahillon et al., 2022). PCR and RT-PCR amplifications were performed using the AMV reverse transcriptase and Taq polymerase (Promega) in combination with primers listed in Table S1 and S2. Reaction mixes and thermocycler conditions have been described before (Mahillon et al., 2022). For the detection of grapevine viruses and viroids, RT-PCR analyses were conducted as previously described (Kofalvi et al., 1997; De Meyer et al., 2000; Jiang et al., 2009; Terlizzi et al., 2011; Ghanem-Sabanadzovic et al., 2012; Beuve et al., 2013). Amplicons were analyzed by agarose gel electrophoresis.

### 2.7 Thrips identification and transmission assay

Identification of the thrips species was achieved by partial sequencing of the *cytochrome oxidase* (COI) gene. In order to do so, total DNA was extracted from a pool of ten adult thrips collected from the diseased *M. annua*, which was followed by PCR amplification using primers previously published (Folmer et al., 1994; Marullo et al., 2020). Amplicons were cloned and sequenced as described above.

For the transmission assay, six 2-week old healthy *M. annua* seedlings were placed inside an insect-proof cage at a distance of 10 cm from six MerV1-infected *M. annua* plants infested with thrips. The leaves of three bait seedlings were dusted with the pollen of ilarvirus-infected *C. quinoa* plants. Three seedlings were maintained outside the cage as healthy controls. After one month, upper leaves from the bait plants were tested for the viruses by RT-PCR.

## 3 Results

### 3.1 Characterization of a new isolate of MerV1

**Fig. 1:**
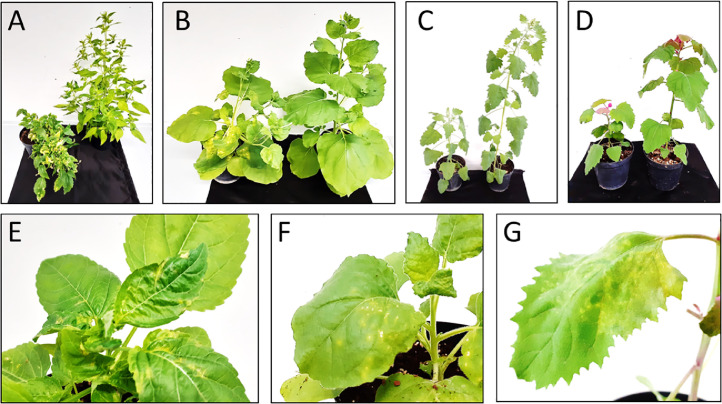
**Symptoms on plants inoculated with a sap produced from the collected *M. annua*. (A-D)** Comparison of an inoculated plant (left) with a healthy control (right), at 30–45 dpi. **(E-G)** Symptoms on upper leaves of infected plants at 15 dpi. A-E: *M. annua*; B-F: *N. benthamiana*; C: *C. quinoa* and d-G: *C. amaranticolor*.

For several years, symptoms consisting of stunting, leaf deformation, chlorosis and necrosis have been noted on annual mercuries grown in the UNIL glasshouses. These symptoms were reportedly more pronounced during warm and dry periods occurring in the summer. A close examination of the plants revealed the presence of thrips, which suggested that a thrips-transmitted viral pathogen might be responsible. In order to test this hypothesis, symptomatic leaves were used for the sap inoculation of healthy *M. annua* seedlings and several indicator species. Inoculated annual mercuries developed local chlorotic spots at 11 days post inoculation (dpi), and reproduced eventually the initially observed systemic symptoms (Fig. 1A and E). Plants of *N. benthamiana* developed local chlorotic spots at 5 dpi, which was followed by systemic symptoms of stunting, deformation and chlorotic spots turning necrotic (Fig. 1B and F). Plants of *C. quinoa* and *C. amaranticolor* developed similar symptoms, consisting of local chlorotic lesions at 3–4 dpi followed by stunting and chlorotic mottling on upper leaves (Fig. 1C, D and G). Local necrotic spots were visible on *N. tabacum* and *N. occidentalis*, but no systemic symptom appeared on these species.

**Fig. 2:**
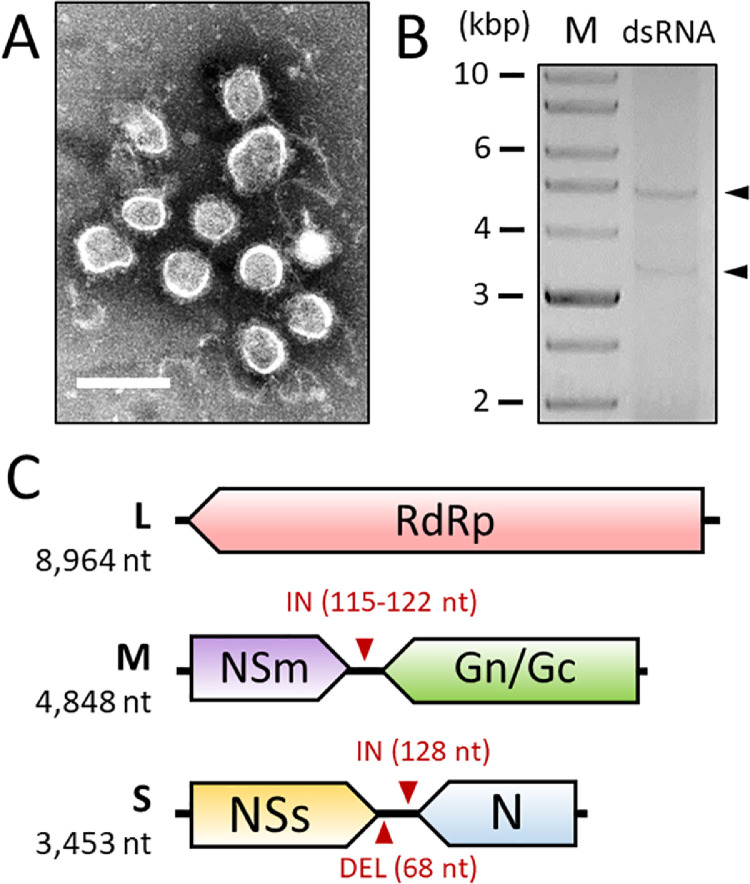
**Characterization of MerV1-CH particles and genome. (A)** Electron micrograph of irregular-to-spherical structures negatively stained with phosphotungstate. The bar represents 100 nm. **(B)** Double-stranded RNA (dsRNA) extracted from sap-inoculated leaves of *N. benthamiana*. Numbers on the left indicate molecular weights (expressed in kilobase pairs - kbp) of a 10 kbp DNA ladder (M). The black arrows indicate the replicative dsRNA intermediates of MerV1-CH segments M and S. **(C)** Schematic representation of MerV1-CH genomic segments. Colored arrows represent ORFs. Small red arrows indicate the positions of large insertions (IN) and deletions (DEL) with regards to the Slovenian isolates. RdRp: RNA-dependent RNA polymerase; NSm: non-structural protein of segment M; Gn/Gc: N and C glycoproteins; NSs: non-structural protein of segment S; N: nucleocapsid protein.

Given the observed symptoms and the presence of thrips, infection by an orthotospovirus was suspected. TEM analyses of *M. annua* leaf-dip preparations revealed the presence of irregularly-shaped spherical structures *c.* 80 nm in diameter which resembled orthotospoviral virions (Fig. 2A). Similar structures were also visible in extracts from *N. benthamiana*, but not from the two *Chenopodium* species (data not shown).

Two low-yield dsRNA elements were extracted from the upper leaves of sap-inoculated *N. benthamiana* plants (Fig. 2B), which lengths were consistent with the replicative intermediates of the two smallest segments of an orthotospovirus. HTS analysis of the dsRNA led to the assembly of three viral contigs (coverages: 4343–19,313 x) sharing extensive similarities with the four MerV1 isolates identified in Slovenia (> 85% nt id., Table S3). The genome of this new MerV1 isolate, hereafter named “MerV1-CH”, was completed by Sanger sequencing and the full-length sequences of the large (L), medium (M) and small (S) segments have been deposited on GenBank under accession numbers PP002150-PP002152, respectively.

The conserved orthotospoviral motif AGAGCAAU is found at the 5′ and 3′ termini of each MerV1-CH segments, confirming a complete sequencing (Herath et al., 2020). The ORFs previously identified for the Slovenian isolates are found on MerV1-CH genome as well (Table S4 and Fig. 2C). Similar to other members of the genus, the intergenic regions in MerV1-CH segments M and S are AT-rich and fold into stable secondary structures (Fig. S1). Notably, these regions exhibit large deletions and insertions with regards to the Slovenian sequences (Fig. 2B), and a comparison of pairwise nucleotide identities clearly indicates that MerV1-CH represents a distinct isolate in the species (Fig. S2).

### 3.2 Characterization of a novel ilarvirus

While MerV1 was detected by RT-PCR in symptomatic plants of *M. annua* and *N. benthamiana*, plants of *C. quinoa* and *C. amaranticolor* did not test positive, suggesting the presence of another pathogen. Strikingly, a second +ssRNA virus was identified in the HTS data of *N. benthamiana* leaves. Indeed, ten contigs (lengths: 206–915 bp; coverages: 22–228 x) showed similarities to sequences associated with several members of the genus *Ilarvirus* (data not shown). The presence of this virus was confirmed by RT-PCR in the upper leaves of the collected *M. annua* as well as in the sap-inoculated plants of *M. annua, N. benthamiana, C. quinoa* and *C. amaranticolor*, indicating that it might have caused the symptoms observed on the two *Chenopodium* species. On the other hand, several RNA samples from asymptomatic plants of *M. annua* also tested positive, and several samples from symptomatic plants tested negative. Altogether, there was no strict association between this ilarvirus and symptoms on annual mercuries, and the name “Mercurialis latent virus” (MeLaV, or MeLaV-CH when compared to other isolates) is therefore used hereafter.

**Fig. 3:**
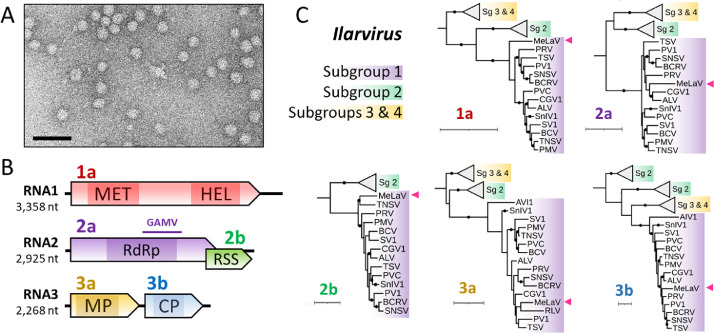
**Characterization of MeLaV particles and genome. (A)** Electron micrograph of MeLaV particles negatively stained with phosphotungstate. The black bar represents 100 nm. **(B)** Schematic representation of MeLaV genomic RNA1, 2 and 3. Colored arrows represent ORFs. Conserved domains are highlighted by shaded areas. See main text for a full description. The region matching GAMV sequence is highlighted by a horizontal bar. **(C)** ML phylogenetic trees for the proteins encoded by members of the *Ilarvirus* subgroups 1–4. The tree scales represent one substitution per site. Black circles on branch indicate bootstrap support > 75 %. The position of MeLaV is highlighted by a pink arrow. Selected members of the genera *Cucumovirus* and *Anulavirus* were used to root the initial trees, except for the 2b tree for which a midpoint rooting was chosen. Complete virus names and accession numbers are listed in Table S6.

In line with previous characterization of ilarviral virions (Pallas et al., 2013; Jaspars, 1999), quasi-spherical particles *c.* 30 nm in diameter were visible in leaf samples from MeLaV-infected *C. amaranticolor* (Fig. 3A). Following RT-PCR amplification and subsequent sequencing of the gaps and termini, three complete genomic RNAs (RNA1–3) were compiled for the virus, the sequences of which have been deposited on GenBank under accession number PP002153-PP002155.

The configuration of the tri-segmented genome of MeLaV is similar to that of other ilarviruses (Fig. 3B). Extensive nucleotide identity is observed in the RNA termini (Fig. S3), and the 3′-UTRs are predicted to form stable structures (Fig. S4), mirroring other members of the genus (Orfanidou et al., 2021). MeLaV RNA1 encodes a protein 1a of 1077 aa which harbors the two following domains: MET (pfam01660, E-value = 1.67e-45) and HEL (pfam01443, E-value = 6.07e-46). RNA2 contains a long ORF followed by a smaller, partially-overlapping ORF. The former encodes a protein 2a of 823 aa that exhibits an RdRp domain (pfam00978, E-value = 6.36e-88), and the latter encodes the RSS 2b of 201 aa. RNA3 contains two non-overlapping ORFs encoding a MP (3a, 303 aa) a CP (3b, 226 aa). These proteins harbor a *Bromovirus* MP domain (pfam01573, E-value = 7.75e-61), and an *Ilarvirus* CP domain (pfam01787, E-value = 2.31e-54), respectively.

The genus *Ilarvirus* is currently divided into four (Rojas and Gilbertson, 2008; Bragard et al., 2013; Alcalá-Briseño et al., 2020; Rivarez et al., 2023a) main subgroups (Pallas et al., 2013). In ML trees built for each protein, MeLaV consistently clusters with members of the subgroup 1 (Fig. 3C). Although the International Committee on Taxonomy of Viruses (ICTV) has not yet defined any threshold for species demarcation among ilarviruses, MeLaV should be considered as a new species considering the low aa identities with other ilarviral homologs. Specifically, MeLaV RdRp and CP (the genes usually chosen for demarcation criteria among plant viruses) only share 63 % and 60 % aa identity with their closest homolog, i.e.*,* the RdRp (QSE03525.1) and CP (AAX47096.1) of TSV, respectively.

### 3.3 Biological characterization of MerV1 and MeLaV

The respective host ranges of MerV1 and MeLaV were assessed by sap inoculations of plant species belonging to nine families (Table S5). Besides *M. annua* and *N. benthamiana*, MerV1 did not induce symptoms on the upper leaves of any other tested species. Local symptoms were visible on sugar beet (*Beta vulgaris*), *N. tabacum* cv. Xanthi, *N. occidentalis* and *C. amaranticolor*. MeLaV induced visible systemic symptoms on *C. quinoa* and *C. amaranticolor*, and it was found to asymptomatically infect *M. annua, N. benthamiana* and two *N. tabacum* cultivars (Xanthi and White Burley).

We hypothesized that the thrips found on the annual mercuries had played a role in the transmission of the viruses in the UNIL glasshouses. Ten adult thrips were collected and exhibited the typical morphology of *Thrips tabaci* (data not shown). This identification was confirmed by sequencing of a 653-bp portion of the COI gene (acc. number: OR976066), revealing only two single nucleotide polymorphisms (SNPs) with GenBank entries corresponding to *T. tabaci* (e.g. MN036454.1 - *T. tabaci* “haplotype 3″ collected in China). This thrips species has been described as a vector for many orthotospoviruses belonging to the IYSV clade, which are presumably transmitted in a circulative, propagative manner. In parallel, the same species is known to facilitate the mechanical inoculation of infected pollen in the case of several ilarviruses (Sdoodee and Teakle, 1993; Bag et al., 2015; Hassani-Mehraban et al., 2019; Greber et al., 1991).

We found that healthy seedlings of *M. annua* became infected with MeLaV and MerV1 when placed in insect-proof cages in proximity to the thrips-infested plants collected from the UNIL glasshouses. Interestingly, while MerV1 infections were maintained in these cages for a year by regularly adding new annual mercuries, MeLaV was no longer present as indicated by negative RT-PCR results. We suspected that MerV1 was efficiently maintained by the thrips, while MeLaV was lost due to the absence of infected pollen. Indeed, the plants placed in the cages would often die before reaching the flowering stage, thereby breaking the transmission cycle.

We detected MeLaV in the pollen produced by sap-inoculated plants of *C. quinoa* (Fig. S5A), which prompted us to use it for a transmission assay. To this end, we placed six healthy *M. annua* seedlings in a new insect-proof cage together with six MerV1-infected plants infested with *T. tabaci*, and we gently dusted the leaves of three of the seedlings with MeLaV-infected pollen from *C. quinoa*, following the examples of transmission studies involving other ilarviruses (Sdoodee and Teakle, 1993; Klose et al., 1996; Greber et al., 1991). After one month, all six bait mercuries were infested by thrips and reproduced the initially-described symptoms. We detected MerV1 in the upper leaves of all six bait plants, and MeLaV was detected in the upper leaves of one of the pollen-dusted plants (Fig. S5B), indicating viral transmission. These preliminary results strongly support the idea that *T. tabaci* has been responsible for the spread of both MerV1 and MeLaV in the UNIL glasshouses.

### 3.4 Previous characterization of MeLaV as GAMV

Surprisingly, a BlastN search on MeLaV genomic RNAs unveiled a striking similarity between a 381 bp region of RNA2 (positions: 1458–1838, Fig. 3B) and the sequence associated with grapevine angular mosaic virus (GAMV, AY590305.1). Specifically, there were only eight SNPs separating the two sequences, corresponding to 100 % id. at the aa level. It is worth mentioning that the sequence of GAMV was obtained in the early 2000s by RT-PCR using degenerate primers, which may explain the SNPs as they are found on the 5′ and 3′ primer binding sites (Girgis et al., 2009; Girgis et al., 2000). The virions associated with GAMV closely resemble those described here for MeLaV. Furthermore, both viruses induce similar symptoms on *C. quinoa*, and pollen-transmission of GAMV was also demonstrated on this plant species (Girgis et al., 2009).

**Fig. 4:**
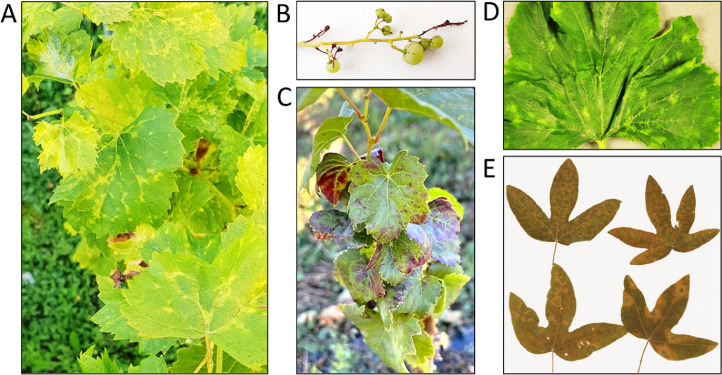
**Grapevines maintained at Agroscope, and leaf samples analyzed at NIVIP. (A)** Grapevine exhibiting AM and grafted onto the “SO4” rootstock. The picture was taken in July 2021. **(B)** Berries produced in 2022 by the plant shown in A. **(C)** Plant of Gamay rouge de la Loire grafted on AM-affected plant and showing leaf curling and red coloration but no AM. The picture was taken in July 2022. **(D)** Leaves of zucchini in which MeLaV-33,558,588 was identified without any other virus. **(E)** Leaves of curuba in which MeLaV-WAG0452486 was identified in co-infection with three other viruses.

Plants showing AM and presumably infected by GAMV were kindly given to Agroscope and NIVIP in previous years. At Agroscope, canes grafted onto the “SO4” rootstock still exhibit the originally-described AM symptoms, i.e., angular chlorotic pattern on leaf blades and leaf deformation (Fig. 4A). In addition, affected plants show flower abortion and produce rare and reduced berries with partial stem necrosis (Fig. 4B), consistent with the initial description of AM (Girgis et al., 2009). Curiously, symptoms reminiscent of grapevine leafroll viruses are observed on red indicators grafted onto AM-affected plants, while no AM symptom is visible on these plants (Fig. 4C).

With the aim to obtain a full sequence of GAMV, a HTS analysis was performed at Agroscope on total RNA extracted from AM-affected leaves. This analysis yielded a total of 8554,952 reads, revealing the presence of the ampeloviruses grapevine leafroll-associated virus 3 and 4 (GLRaV-3 and 4, with 30,122 and 5784 mapped reads, respectively), the vitivirus grapevine virus A (4954 reads) and the foveavirus grapevine rupestris stem pitting-associated virus (17,161 reads). Three viroids were also identified: hop stunt viroid 1 (174 reads) and grapevine yellow speckle viroid 1 and 2 (30 and 198 reads, respectively). However, no HTS read was found matching GAMV sequence. Individual RT-PCR analyses further confirmed the presence of the aforementioned viruses and viroids in the AM-affected grapevines, while there was no detection of GAMV (Fig. S6). A HTS analysis performed independently at NIVIP on another AM-affected grapevine clone resulted in the identification of the same viruses and viroids, except for the additional detection of grapevine fleck virus but the absence of GLRaV-4 (M.B. and R.S., unpublished). Collectively, these data suggest that MeLaV and GAMV most likely refer to the same virus, and question a role in AM of grapevine.

### 3.5 Identification of MeLaV in The Netherlands

The genomes of three additional MeLaV isolates were obtained by HTS of total RNA from symptomatic leaves, which were analyzed at NIVIP in The Netherlands (Table 1).

**Table 1:** MeLaV isolates detected in symptomatic leaves at NIVIP in The Netherlands.

**Isolate**	Original host*	**Location**	**Year**	**Total reads**	**Mapped reads**	**RNA**	**SNPs**⁎⁎	**Size (nt)**	**Access.**
WAG0452486	*P. tripartita* var. *mollissima*	NL	1991	40,302,348	92,948	1	24	3165	PP002143
					42,629	2	23	2908	PP002146
					80,306	3	12	2256	PP002149
6,934,090	*C. pepo*	Limburg, NL	2017	13,788,978	146,458	1	16	3352	PP002141
					97,252	2	12	2917	PP002144
					497,327	3	14	2263	PP002147
33,558,588	*C. pepo*	Almeria, ES	2018	41,631,798	743,960	1	16	3352	PP002142
					339,243	2	21	2917	PP002145
					2473,324	3	16	2263	PP002148

⁎ Isolates 6,934,090 and 33,558,588 were sequenced following sap inoculations on cucumber and zucchini, respectively.

⁎⁎ Single nucleotide polymorphisms with the respective RNAs of MeLaV-CH, excluding gaps.

These three genomes are near complete and are highly similar to that of MeLaV-CH (> 99.2% nt id.). As MeLaV-6,934,090 was found in a zucchini leaf showing irregular yellow spots in mixed infection with watermelon mosaic virus, the pathogenicity of this isolate is unclear. In contrast, no other virus was found with MeLaV-33,558,588 identified in a zucchini leaf showing irregular chlorotic spots and slight vein-banding (Fig. 4D), providing strong evidence that MeLaV can induce symptom on this plant species. Remarkably, MeLaV-WAG0452486 was identified in historical samples of curuba leaves exhibiting chlorotic rings and spots (Fig. 4E), which have been conserved since 1991 in a herbarium collection at Wageningen (The Netherlands). In this case, the virus was found in mixed infection with Passiflora chlorosis virus, Passiflora latent virus and potato virus S. Altogether, these results suggest that MeLaV can infect and produce symptoms on zucchini and possibly curuba, which further expands its putative host range.

### 3.6 Detection of MeLaV in SRA datasets

In order to look for potential traces of MerV1 and MeLaV in public RNA sequencing depositories, we used the palmID tool of Serratus that can detect viral RdRp motifs in NCBI SRA runs. While only distant homologs were yielded for MerV1 (data not shown), this search revealed 31 RdRp-containing contigs representing fragments of MeLaV RNA2 in various datasets.

Twenty-one viral fragments, detailed in Table S7, are associated with the transcriptomic analyses of healthy annual mercuries conducted at UNIL in Lausanne in 2017–2018 (Cossard et al., 2019; Veltsos et al., 2019). The remaining MeLaV RNA2 fragments are present in HTS datasets from 2013 to 2020 associated with diverse species in Switzerland and four other European countries (Table 2). One fragment is found in a transcriptomic study of the closely related species *Mercurialis huetii*, which was undertaken by the same laboratory in 2017 (J. R. Pannell, unpublished). Four fragments are identified in transcriptomes of the Crete arum (*Arum concinnatum*) in Greece, and individual HTS datasets from the carnivorous Venus flytrap (*Dionaea muscipula*) in Germany and the bread wheat (*Triticum aestivum*) in France (Iosip et al., 2020; Choulet et al., 2014; Onda et al., 2015). Notably, fragments of MeLaV RNA2 are also identified in three metaviromics analyses of honey bees in Belgium (Deboutte et al., 2020).

**Table 2:** Fragments of MeLaV RNA2 identified in SRA datasets.

**Host**	**Location**	**Year**	**Tissue**	**Size (nt)**	Cover*	**SRA run**
*A. mellifera*	Flanders, BE	2013	Whole body	1508	5.7	SRR10418344
				1500	1.0	SRR10418332
				1525	12.8	SRR10418312
*A. concinnatum*	Panormos, Crete, GR	2013	Male florets	1467	8.5	DRR023772
			Female florets	596	0.2	DRR023776
			Appendix	1472	38.9	DRR023774
			Female florets	1467	11.1	DRR023773
*T. aestivum*	FR	2014	Stem	798	0.1	ERR424762
*M. huetii*	Lausanne, CH	2017	Flower buds and leaf	1466	4.7	SRR5219180
*D. muscipula*	Würzburg, DE	2020	Trigger hair	1465	1.0	ERR4508087

⁎ Expressed in contig coverage per million of total reads. Note that RNA extraction and sequencing methods are variable among the SRA runs.

**Fig. 5:**
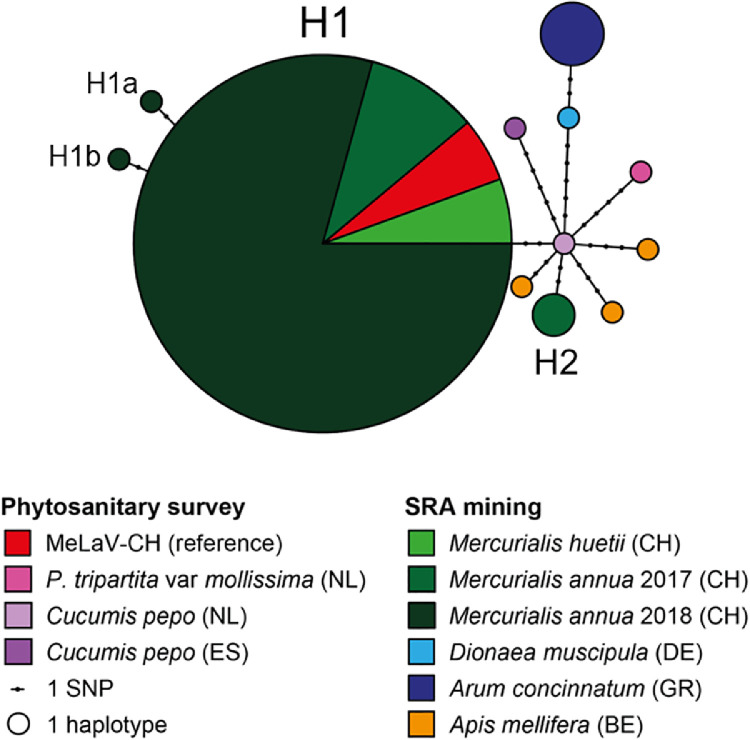
**Haplotype network for MeLaV isolates.** The network is based on a trimmed alignment of long (> 1 kb) fragments of the RNA2. Sequences have been either gathered through phytosanitary surveys at Agroscope (MeLaV-CH) and NIVIP (Table 1) or through SRA mining (Table 2). Each circle corresponds to a haplotype, the size of which is proportional to the number of sequences. Each dot represents a single nucleotide polymorphism (SNP) separating two haplotypes.

The diversity of the aforementioned sequences was evaluated with a haplotype network based on the trimmed alignment of long RNA2 fragments (Fig. 5). Based on this analysis, we observed a low inter-sequence diversity, with only 11 SNPs (out of 1027 positions) separating the most divergent haplotypes. This low diversity mirrors recent data obtained for the other ilarvirus SlnV1 also identified in numerous HTS datasets (Rivarez et al., 2023b). Hence, the virus seems to be quite stable on different plant species and over a relatively long period of time. The major MeLaV haplotype, dubbed “H1”, corresponds to MeLaV-CH and most isolates associated with the *M. annua* transcriptomic analyses in Lausanne. Interestingly, it seems that H1 was already present in 2017 in the UNIL glasshouses together with the minor haplotype “H2”. After 2017, H2 was lost while H1 became dominant, producing hypothetically the two sub-haplotypes “H1a” and “H1b”. The network does not provide any other clear association among the haplotypes, besides a proximity between the sequences identified in the Venus flytrap and Crete arum.

## 4 Discussion

The results obtained from sap inoculations, TEM and HTS analyses showed that the annual mercuries grown at the UNIL glasshouses were infected with MerV1 and MeLaV. While the former was clearly associated with symptoms, the latter was found to be latent and has been unnoticed in the university glasshouses since at least 2017, infecting multiple plants used for research on the complex *M. annua* sexual systems (Cossard et al., 2019; Veltsos et al., 2019). These invisible infections mirror recent results obtained for Arabidopsis latent virus 1 (ArLV1), a seed-transmissible comovirus infecting numerous laboratory accessions of *Arabidopsis thaliana*, which has been similarly unnoticed for many years (Verhoeven et al., 2023). Although ArLV1 is asymptomatic on *A. thaliana*, it has been associated with noticeable changes including an improved drought tolerance, which may thus impact studies unaware of this invisible parasite (Verhoeven et al., 2023). Likewise, future research involving *M. annua* should take into account the potential infection by MeLaV and its unknown physiological impact.

The sources of introduction of MeLaV and MerV1 in the UNIL glasshouses are unknown. It is imaginable that both viruses were infecting wild hosts in the vicinity of the university buildings, and the year-round production of annual mercuries in glasshouse had offered a suitable environment for the establishment of *T. tabaci* colonies and subsequent inoculation and maintenance of both viruses. It is also possible, although less likely, that these agents were first imported via infected seeds, as documented for several ilarviruses and tospoviruses (Pallas et al., 2012; Groves et al., 2016). In fact, MeLaV was shown to be seed-transmissible in *C. quinoa* when characterized as GAMV (Girgis et al., 2009), and this might also be the case in *M. annua*.

This study represents the second record of MerV1 in Europe, in line with the idea that orthotospoviruses from the IYSV clade have an Eurasian origin (Hassani-Mehraban et al., 2005). Notably, viruses from this group have the potential to emerge as important crop pathogens. In particular, IYSV was initially described in The Netherlands in 1992 (Cortês et al., 1998), and became economically problematic during the 2000s, causing outbreaks in many onion crops worldwide (Bag et al., 2015). Other members of the clade have broad host ranges that include important crop species (Hassani-Mehraban et al., 2005; Ciuffo et al., 2008; Dong et al., 2013), so that additional evaluation of MerV1 pathogenicity is needed.

Based on phylogenetic analyses, MeLaV should be considered as a new species of the *Ilarvirus* subgroup 1. It seems that this virus can infect species from three families (*Amaranthaceae, Euphorbiaceae* and *Solanaceae*), and additional hosts are suggested by phytosanitary surveys and transcriptomic data. In particular, MeLaV contigs in multiple HTS datasets of zucchini and Crete arum likely reflect true infections. On the other hand, contigs in individual datasets (e.g. samples from France and Germany) are questionable. It is possible that these contigs represent contaminations, which may have occurred at any steps from the sampling to the sequencing. In particular, they might correspond to contaminating MeLaV-infected pollen, as hypothesized for SlnV1 (Rivarez et al., 2023b). The presence of MeLaV contigs in several metaviromics datasets of bees in Belgium conforms with the idea that this virus can be detected through infected pollen, and argues that pollinators disseminate this virus as demonstrated for several other ilarviruses.

We suspect that the initial characterization of MeLaV as GAMV may have arisen through pollen contamination. This scenario has been also hypothesized for SlnV1, which was detected in grapevine by HTS and RT-PCR but later found to likely not infect this species (Rivarez et al., 2023b). It is conceivable that GAMV-infected pollen grains were inadvertently introduced during the preliminary work on AM, possibly residing on the surfaces of grapevine leaves, and subsequently transmitted via sap inoculation. This is supported by the fact that “the virus was isolated with difficulty in 1 out of 50 inoculated *Gomphrena globosa*” using a grapevine sap (Girgis et al., 2009). The source of infected pollen may have been wind-pollinated anemophilous wild plants such as annual mercuries. Nonetheless, while GAMV is clearly not linked to AM, the initial identification may have represented a true infection. It is possible that this virus was unevenly distributed within the grapevine tissues, a trait previously observed for ilarviruses in perennial hosts (Thomas-Sharma et al., 2018). The virus might have been lost through the production of cuttings, and thus not detected through our HTS and RT-PCR analyses. However, we did not find HTS read matching MeLaV/GAMV in any grapevine-associated SRA datasets, which brings further support for the pollen contamination hypothesis.

The cause of AM of grapevine remains unclear. Although a complex interaction between the detected grapevine viruses and viroids cannot be ruled out, the same virus cocktail did not induce AM to red indicators. We are currently gathering more genomic and biological data on these agents, which will hopefully help to assess their putative involvement in AM. A genetic origin can also be hypothesized for this disorder since the affected grapevines have been obtained after self-pollination (Girgis et al., 2009).

The widespread distribution of MeLaV in Europe calls for additional evaluation of its pathogenicity. In particular, future studies should address the possibility of synergism with other viruses such as MerV1 and the co-infecting viruses identified in The Netherlands. Additional transmission studies are also needed as *T. tabaci* has a worldwide distribution, wide host range and can readily invade crop fields and glasshouses (Loredo Varela and Fail, 2022). Importantly, it should be noted that ilarviruses and tospoviruses can use multiple thrips species as vector. Besides *T. tabaci*, other thrips have been described on *M. annua* (Atakan et al., 2013; Atakan, 2008), and it would be interesting to test whether these species can transmit MeLaV and MerV1 too.

## 5 Conclusion

Our analyses showed that MeLaV is a new tri-segmented +ssRNA virus belonging to the *Ilarvirus* subgroup 1. The virus is widespread in Europe, where it likely circulates in a typical ilarviral manner, which requires the production of infected pollen followed by a thrips-mediated mechanical inoculation. MeLaV is also sap transmissible, and is probably vertically transmitted in *C. quinoa* (Girgis et al., 2009). In terms of pathogenicity, while the virus latently infects *M. annua* and several other species, it induces symptoms on two *Chenopodium* species and probably on the economically important *C. pepo*. There are compelling evidences suggesting that MeLaV is synonymous to GAMV, but this virus is likely not involved in angular mosaic of grapevine. Hence, we call for the recognition of MeLaV by the ICTV, and the removal of GAMV from the long list of symptomatic grapevine viruses (Fuchs, 2020). Consistent with recent findings obtained for SlnV1, our study emphasizes that ilarvirus-infected pollen can be a confusing source of contamination during HTS and other diagnostic procedures, *a fortiori* in the context of latent infections.

## Funding

This research did not receive any specific grant from funding agencies in the public, commercial, or not-for-profit sectors.

## Ethical approval

This article does not contain any studies with human participants or animals requiring ethical approval.

## CRediT authorship contribution statement

**Mathieu Mahillon:** Conceptualization, Investigation, Formal analysis, Visualization, Data curation, Writing – original draft. **Justine Brodard:** Investigation, Resources, Data curation, Writing – review & editing. **Ruben Schoen:** Investigation, Resources, Writing – review & editing. **Marleen Botermans:** Investigation, Resources, Writing – review & editing. **Nathalie Dubuis:** Investigation, Methodology. **Raphaël Groux:** Software, Writing – review & editing. **John R. Pannell:** Resources, Writing – review & editing. **Arnaud G. Blouin:** Conceptualization, Supervision, Writing – review & editing. **Olivier Schumpp:** Supervision, Project administration, Writing – review & editing.

## Declaration of competing interest

The authors declare that they have no known competing financial interests or personal relationships that could have appeared to influence the work reported in this paper.

## Data Availability

No data was used for the research described in the article.

## Appendix Supplementary materials

Image, application 1
