# Supplementary material for: Revisiting a pollen-transmitted ilarvirus previously associated with angular mosaic of grapevine
Source: Virus Res. 2024 Mar 22;344:199362. doi: 10.1016/j.virusres.2024.199362 (PMC10979282; doi:10.1016/j.virusres.2024.199362)
Supplement: Supplementary file 1 [file mmc1.docx]

**Supplementary figures**

**
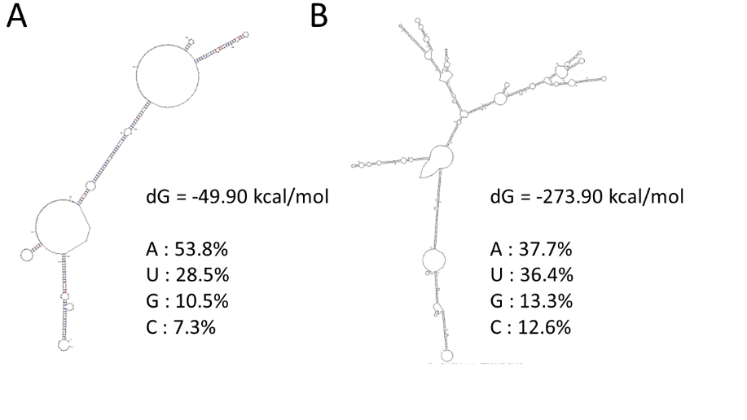
**

**Figure S1. Secondary structures predicted for the intergenic regions of MerV1-CH segments M (A) and S (B).** The minimum optimal energy (dG) and nucleotide composition (in percent) are given.


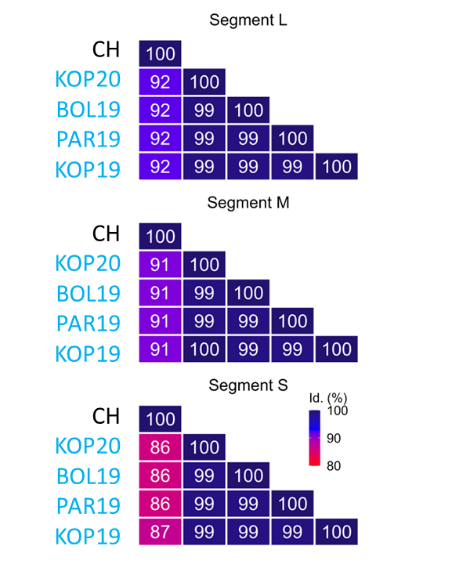


**Figure S2. Pairwise nucleotide identities for the three segments of MerV1 isolates.** The identities (in percent) are determined based on a trimmed alignment, excluding gaps.


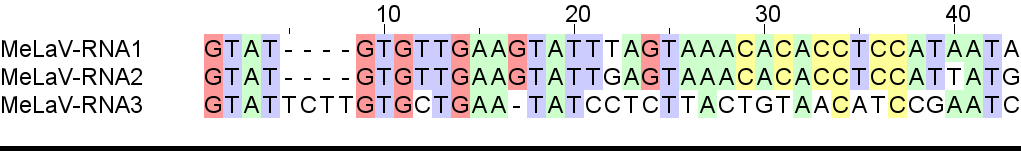


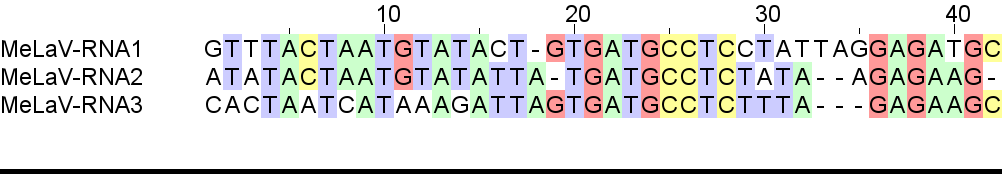


**Figure S3. Alignment of the termini of MeLaV RNAs.** Up: 5’-termini. Down: 3’-termini. Colored background indicates conservation in at least two sequences.


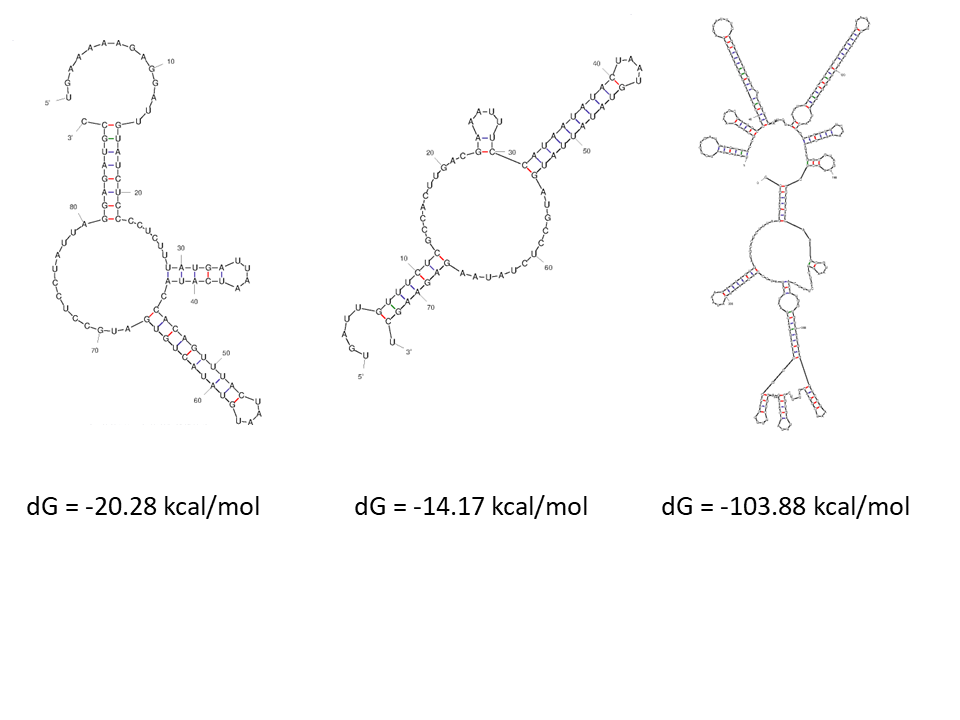


**Figure S4.** **Secondary structures predicted for the 3’-UTRs of MeLaV.** Left, center and right panels show the structures predicted for the RNA1, 2 and 3, respectively. The minimum optimal energy (dG) is given for each structures.


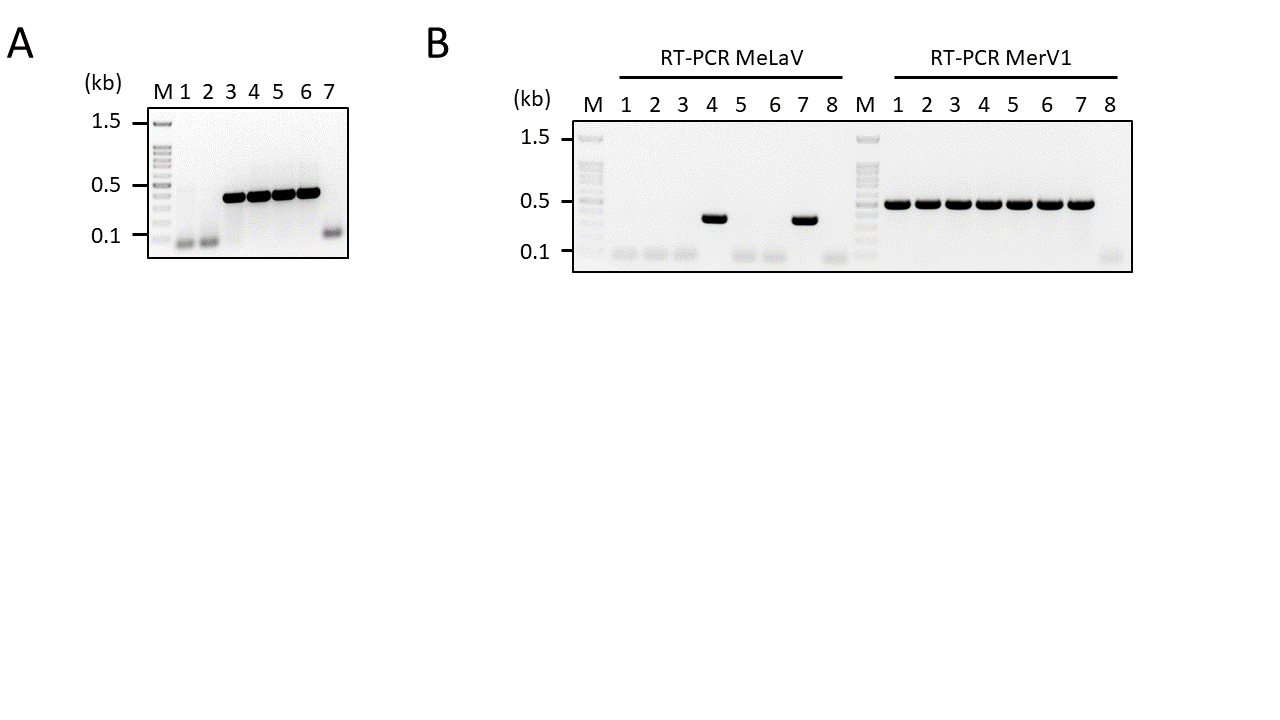


**Figure S5. Transmission of MeLaV and MerV1**. **(A)** RT-PCR detection of MeLaV in RNA samples extracted from *C. quinoa*. Samples are as follows; 1-2: pollen from healthy plants; 3-5: pollen from MeLaV-infected plants; 6: positive control – RNA sample from MeLaV-infect leaf; 7: RT-PCR negative control - water. **(B)** RT-PCR detection of MeLaV (left) and MerV1 (right) in RNA samples from the upper leaves of *M. annua* as follows; 1-3: non-dusted bait plants; 4-6 bait plants dusted with MeLaV-infected pollen; 7: positive control - a co-infected plant; 8: negative control - water. For both gels, M: 1 kb ladder DNA ladder. Numbers on the left are chosen DNA weights expressed in kilobase (kb).


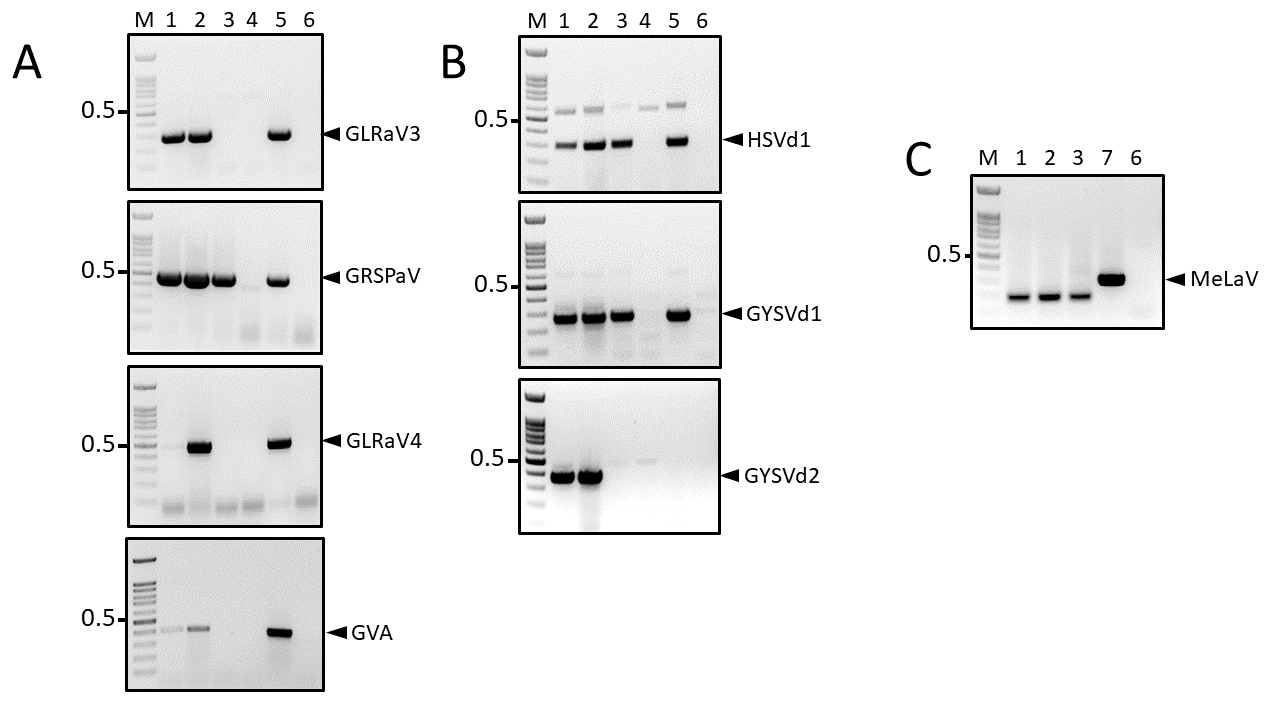


**Figure S6. RT-PCR detections of the viruses and viroids infecting AM-affected grapevines. (A)** Detection of viruses. **(B)** Detection of viroids. **(C)** Detection of MeLaV/GAMV. Samples are as follows; 1: grapevine affected with AM; 2: Cutting from plant 1; 3: positive control for GRSPaV; 4: healthy non-infected grapevine; 5: positive control: GLRaV3, GLRaV4, GRSPaV and GVA; 6: PCR negative control (water); 7: MeLaV-infected annual mercury. GLRaV-3: grapevine leafroll-associated virus 3; GLRaV-4: grapevine leafroll-associated virus 3; GRSPaV: grapevine rupestris stem pitting-associated virus; GVA: grapevine virus A; HSVd1: Hop stunt viroid and GYVd1-2: Grapevine yellow speckle viroid 1 and 2.

**Supplementary tables**

**Table S1. Primers used in this study.**

| **Name** | **Sequence** | **Use** |
| --- | --- | --- |
| NonoIlarFor | CCGACTGGGAAATACCACCA | RT-PCR detection of MeLaV RNA2 |
| NonoIlarRev | CATCACCACTGGCAGTCACA |  |
| GAMVFOR | GTAAGGTGTTTTCTATGTTGGGACT | RT-PCR detection of MeLaV/GAMV |
| GAMVREV | ACAAAGATACACACAATTCCTCCT |  |
| TOSPONOD1F | TGAAGCGTGTCTGTATCCCG | RT-PCR detection of MerV1 |
| TOSPONOD1R | GTGCGATGATTCTGTGCAGC |  |
| Race5Tosp1 | GCGAAGGTTGCAGCACTGCATGAATTCC | 5’ sequencing of MerV1 segment L |
| Race5Tosp1Nested | CCTTGAAACACGATATTGTAAC |  |
| Race3Tosp1 | TCCATTGACATAGTGCTCTGGT | 3’ sequencing of MerV1 segment L |
| Race5Tosp2 | CTCCTCCTGCTGTCCCATGTAGGCATCAGAC | 5’ sequencing of MerV1 segment M |
| Race5Tosp2Nested | TGCTTTGTTCAATCACAAGCCT |  |
| Race3Tosp2 | CAATACCATACGGCACTGTGA | 3’ sequencing of MerV1 segment M |
| Race5Tosp3 | GCCATTAGAGGAAGAGGACCTCCCAGACTC | 5’ sequencing of MerV1 segment S |
| Race5Tosp3Nested | AAGTCAGATGGTCCAGCCAC |  |
| Race3Tosp3 | CCTTGACAATAGACATGCTTG | 3’ sequencing of MerV1 segment S |
| Race5Ila1 | GGGACTTCCACTCCTCATCGTTACCCCG | 5’ sequencing of MeLaV RNA1 |
| Race5Ila1Nested | AGTCACCCCATCTGTCATCC |  |
| Race3Ila1 | AGGTGACACTTGGGATCATGT | 3’ sequencing of MeLaV RNA1 |
| Race5Ila2 | CGGAACAAATCGGCCCAAAGCCAATTCC | 5’ sequencing of MeLaV RNA2 |
| Race5Ila2Nested | GCCCAAAGCCAATTCCAAGG |  |
| Race3Ila2 | AGGTTACAATTGAAGAGTTTGGGA | 3’ sequencing of MeLaV RNA2 |
| Race5Ila3 | GTACCACCATAACATTCTTCACCCGTAGCC | 5’ sequencing of MeLaV RNA3 |
| Race5Ila3Nested | GCTTGTGCATGGGTGGTTTT |  |
| Race3Ila3 | GCATCAAAGCGATGCCTACG | 3’ sequencing of MeLaV RNA3 |

**Table S2** Primers for the RT-PCR detection of grapevine viruses and viroids identified in AM-affected plants.

| **Targets** | **Primers** | **Sequences** | **References** |
| --- | --- | --- | --- |
| GLRaV-3 | LR3-POL-F1 | ACGTAACGGGGCAGAATATAGT | Beuve *et al.* 2013 |
|  | LR3-POL-R1 | TATCAACACCAAGTGTCAAGAGTA |  |
| GRSPaV | RSP35 | AGRYTTAGRGTRGCTAARGC | Terlizzi *et al.* 2011 |
|  | RSP36 | CACATRTCATCVCCYGCAAA |  |
| GLRaV-4 | LR-AmpF | ATTTAGGTAATGTWGTRGCTAC | Ghanem-Sabanadzovica *et al.* 2012 |
|  | LR-AmpR | TATCCTCAGWGAGGAARCGG |  |
| GVA | G1 | ATACTCTCTTCGGGTACATCGC | De Meyer *et al.* 2000 |
|  | G4 | GTGCATGGCCTGTATCACAGT |  |
| HSVd | HSV1 | CGCCCGGGGCAACTCTTCTCAGAATCC | Kofalvi *et al.* 1997 |
|  | HSV2 | GCCCCGGGGCTCCTTTCTCAGGTAAG |  |
| GYSVd1 | GYSVd1-11F | TGCTTGTGGTTCCTGTGGTT | This study |
|  | GYSVd1-283R | AAAGAGACCAAGTCCGCTCG |  |
| GYSVd2 | GYSVd2-P1 | ACTAGTACTTTCTTCTATCTCCGAAGC | Jiang *et al.* 2009 |
|  | GYSVd2-P2 | ACTAGTCCGAGGACCTTTTCTAGCGCTC |  |

**Table S3.** Description of the Slovenian isolates of MerV1.

| **Isolates** | **Segment** | **Size (bp)** | **Accession numbers** |
| --- | --- | --- | --- |
| MerV1-BOL19SW | L | 8,935 | OL471973.1 |
|  | M | 4,706 | OL471974.1 |
|  | S | 3,376 | OL471975.1 |
| MerV1-PAR19SW | L | 9,055 | OL471970.1 |
|  | M | 4,714 | OL471971.1 |
|  | S | 3,382 | OL471972.1 |
| MerV1-KOP19SW | L | 8,946 | OL471967.1 |
|  | M | 4,708 | OL471968.1 |
|  | S | 3,378 | OL471969.1 |
| MerV1-KOP20SW | L | 8,976 | OL471964.1 |
|  | M | 4,727 | OL471965.1 |
|  | S | 3,392 | OL471966.1 |

**Table S4.** ORFs found on MerV1-CH segments and associated domains.

| **Segment** | **ORF** | **Positions (nt)** | **Domain on ORF** | **Positions (aa)** | **E-value** |
| --- | --- | --- | --- | --- | --- |
| L | RdRp | 289-8940 | Endonuclease cd22349 | 834-1673 | 2.41e-146 |
|  |  |  | *Bynyavirus* RdRp pfam04196 | 93-241 | 1.29e-04 |
| M | NSm | 61-993 | MP pfam00803 | 91-279 | 7.59e-13 |
|  | Gn/Gc | 1394-4825 | *Bunyavirus* glycoprotein pfam03557 | 579-1029 | 3.97e-19 |
| S | NSs | 64-900 | *Bunyavirus* NS protein pfam03231 | 4-441 | 2.50e-175 |
|  | N | 2058-3389 | Nucleocapsid pfam01533 | 9-257 | 2.19e-30 |

**Table S5.** Host range of MerV1 and MeLaV based on sap inoculations.

| **Plant family and species** | **MerV1** | | | **MeLaV** | | |
| --- | --- | --- | --- | --- | --- | --- |
|  | **Inoculated leaves** | **Upper**  **leaves** | **RT-PCR*** | **Inoculated leaves** | **Upper**  **leaves** | **RT-PCR*** |
| ***Apocynaceae*** |  |  |  |  |  |  |
| *Catharanthus roseus* | ns | ns | - | ns | ns | - |
| ***Amaranthaceae*** |  |  |  |  |  |  |
| *Beta vulgaris* cv. Agueda | CL | ns | - | ns | ns | - |
| *Chenopodium quinoa* | ns | ns | - | NRS | MO, S | + |
| *Chenopodium amaranticolor* | NS | ns | - | NRS | MO, S | + |
| ***Brassicaceae*** |  |  |  |  |  |  |
| *Brassica rapa subsp. pekinensis* | ns | ns | - | ns | ns | - |
| ***Cucurbitaceae*** |  |  |  |  |  |  |
| *Cucumis sativus* | ns | ns | - | ns | ns | - |
| ***Euphorbiaceae*** |  |  |  |  |  |  |
| *Mercurialis annua* | CL | S, D, CL, NL | + | ns | ns | + |
| ***Lamiaceae*** |  |  |  |  |  |  |
| *Agastache rugosa* | ns | ns | - | ns | ns | - |
| ***Leguminosae*** |  |  |  |  |  |  |
| *Phaseolus vulgaris* | ns | ns | - | ns | ns | - |
| ***Malvaceae*** |  |  |  |  |  |  |
| *Malva sylvestris* | ns | ns | - | ns | ns | - |
| ***Solanaceae*** |  |  |  |  |  |  |
| *Nicotiana benthamiana* | CL | S, D, CL, NL | + | ns | ns | + |
| *Nicotiana clevendii* | ns | ns | - | ns | ns | - |
| *Nicotiana occidentalis* | NL | ns | - | ns | ns | - |
| *Nicotiana tabacum* cv. Xanthi | NL | ns | - | ns | ns | + |
| *Nicotiana tabacum* cv. White burley | ns | ns | - | ns | ns | + |
| *Physalis peruviana* | ns | ns | - | ns | ns | - |
| *Solanum lycopersicum* cv. Moneymaker | ns | ns | - | ns | ns | - |

CL: chlorotic lesion; NL: necrotic lesions, Mo: mottling, NRS: necrotic ringspot, ns: no symptom, + and – refers to the detection of RT-PCR amplicons from RNA sample in upper non-inoculated leaves. * RT-PCR analyses were performed on RNA extracted from upper leaves.

**Table S6.** Accession numbers for the proteins of ilarviruses.

| **Virus** | **Acronym** | **1a** | **RdRp (2a)** | **RSS (2b)** | **MP (3a)** | **CP (3b)** |
| --- | --- | --- | --- | --- | --- | --- |
| Actinidia yellowing ringspot virus |  | QJQ13982.1 | QJQ13983.1 |  | QJQ13984.1 | QJQ13985.1 |
| Ageratum latent virus | ALV | YP_008470969.1 | YP_008470970.1 | YP_008470971.1 | YP_008470972.1 | QNL13312.1 |
| Apple ilarvirus 1 | AIV1 | NA | QIC52823.1* | NA | QIC52824.1 | QIC52825.1 |
| Apple mosaic virus |  | NP_604469.1 | NP_604470.1 | NA | NA | NA |
| Asparagus virus 2 |  | NA | YP_002455929.1 | YP_002455930.1 | NA | NA |
| Bacopa chlorosis virus | BCV | UOF93184.1 | UOF93185.1 | UOF93186.1 | AFD04721.1 | AFD04722.1 |
| Blackberry chlorotic ringspot virus | BCRV | YP_002308569.1 | YP_002308570.1 | YP_002308571.1 | YP_002308572.1 | YP_002308573.1 |
| Cape gooseberry ilarvirus 1 | CGV1 | YP_009551515.1 | YP_009551513.1 | YP_009551514.1 | YP_009551516.1 | YP_009551517.1 |
| Citrus leaf rugose virus |  | NP_613282.1 | NP_613281.1 | NP_619712.1 | NP_613279.1 | NP_613280.1 |
| Citrus variegation virus |  | YP_001285482.1 | YP_001285483.1 | YP_001285484.1 | YP_001285480.1 | YP_001285481.1 |
| Elm mottle virus |  | NP_619576.1 | NP_619575.1 | NP_620071.1 | NP_619577.1 | NP_619578.1 |
| Fragaria chiloensis latent virus |  | YP_164801.1 | YP_164802.1 | YP_164803.1 | YP_164804.1 | YP_164805.1 |
| Grapevine associated ilarvirus | GAV | QIJ25693.1 | QIJ25694.1 | QIJ25695.1 | QIJ25696.1 | QIJ25697.1 |
| Grapevine virus S |  | AFV34763.1 | AFV34764.1 | NA | NA | NA |
| Hydrangea vein-banding virus |  | ULT85592.1 | ULT85593.1 | ULT85594.1 | ULT85595.1 | ULT85596.1 |
| Lilac ring mottle virus |  | NA | YP_009507940.1 | YP_009507941.1 | YP_009507938.1 | YP_009507939.1 |
| Parietaria mottle virus | PMV | YP_006446.1 | YP_006447.1 | YP_006448.1 | YP_006463.1 | YP_006464.1 |
| Peanut virus C | PVC | AWC08303.1 | AWC08304.1 | AWC08305.1 | AWC08306.1 | AWC08307.1 |
| Potato yellowing virus |  | QGL51777.1 | QGL51778.1 | NA | QGL51779.1 | QGL51780.1 |
| Privet ringspot virus | PRV | YP_009165996.1 | YP_009165997.1 | YP_009165998.1 | YP_009165999.1 | YP_009166000.1 |
| Prune dwarf virus |  | YP_611154.1 | YP_611151.1 | NA | YP_611152.1 | YP_611153.1 |
| Prunus necrotic ringspot virus |  | NP_733823.1 | NP_733824.1 | NA | NP_733825.1 | NP_733826.1 |
| Prunus virus 1 | PV1 | UOF93064.1 | UOF93065.1 | UOF93066.1 | UOF93067.1 | UOF93068.1 |
| Raphanus latent virus |  | AER57897.1* | AER57898.1 | NA | AER57899.1 | AER57900.1 |
| Rosa ilarvirus-1 |  | QPB74030.1 | QPB74031.1 | QPB74032.1 | QPB74033.1 | QPB74034.1 |
| Solanum nigrum ilarvirus 1 | SnIV1 | UTQ50863.1 | UTQ50864.1 | UTQ50865.1 | UTQ50866.1 | UTQ50867.1 |
| Soybean ilarvirus 1 | SV1 | UXP11828.1 | UXP11829.1 | UXP11830.1 | UXP11831.1 | UXP11832.1 |
| Spinach latent virus |  | NP_620677.1 | NP_620678.1 | NP_620679.1 | NP_620680.1 | NP_620681.1 |
| Strawberry necrotic shock virus | SNSV | YP_941474.2 | YP_941472.1 | YP_941473.1 | YP_941470.1 | YP_941471.1 |
| Tobacco streak virus | TSV | NP_620772.1 | NP_620768.1 | NP_620769.1 | NP_620773.1 | NP_620774.1 |
| Tomato necrotic spot virus | TNSV | AYN45099.1 | AYN45100.1 | AYN45101.1 | AYN45102.1 | AYN45104.1 |
| Cucumber mosaic virus |  | AVI01376.1 | AVI01377.1 | NA | AVI01379.1 | AVI01380.1 |
| Tomato aspermy virus |  | NP_620760.1 | NP_620761.1 | NA | NP_620758.1 | NP_620759.1 |
| Grapevine line pattern virus |  | UIO53002.1 | UIO53001.1 | NA | UIO53000.1 | QKI29192.1 |
| Pelargonium zonate spot virus |  | NP_619770.1 | NP_619771.1 | NA | NP_619772.1 | NP_619773.1 |

***Only partial sequence.**

**Table S7.** SRA runs of *M. annua* transcriptomes in which MeLaV RNA2 fragments were identified.

| **BioProject** | **SRA run** | **Biosample** | **Host** | **Stage** | **Tissue** | **Cover** | **Year** |
| --- | --- | --- | --- | --- | --- | --- | --- |
| PRJNA369310 | SRR5219173 | SAMN06284066 | Male M2 | Sexually mature | Flowers and leaves | 152,69 | 2017 |
|  | SRR5219176 | SAMN06284063 | Female G2 | Sexually mature | Flowers and leaves | 70,74 |  |
|  | SRR5219124 | SAMN06284115 | Female fO | Sexually mature | Flowers and leaves | 15,47 |  |
|  | SRR5219120 | SAMN06284119 | Male mC | Sexually mature | Flowers and leaves | 27,09 |  |
|  | SRR5219145 | SAMN06284094 | Female fA | Sexually mature | Flowers and leaves | 61,59 |  |
| PRJEB26963 | ERR2597551 | SAMEA4689065 | Diploid male | Sexually mature | Leaf | 12,63 | 2018 |
|  | ERR2597549 | SAMEA4689063 | Diploid female | Sexually mature | Leaf | 7,94 |  |
|  | ERR2597570 | SAMEA4689084 | Diploid female | Sexually mature | Root | 23,9 |  |
|  | ERR2597545 | SAMEA4689059 | Diploid male | Sexually mature | Leaf | 5,55 |  |
|  | ERR2597569 | SAMEA4689083 | Diploid female | Sexually mature | Root | 16,15 |  |
|  | ERR2597571 | SAMEA4689085 | Diploid male | Sexually mature | Root | 99,43 |  |
|  | ERR2597552 | SAMEA4689066 | Diploid female | Sexually mature | Leaf | 3,74 |  |
|  | ERR2597568 | SAMEA4689082 | Diploid female | Sexually mature | Root | 186,8 |  |
|  | ERR2597572 | SAMEA4689086 | Diploid female | Sexually mature | Root | 107,4 |  |
|  | ERR2597565 | SAMEA4689079 | Diploid male | Sexually mature | Root | 11,2 |  |
| PRJEB27300 | ERR2639628 | SAMEA4729062 | Diploid female | Sexually mature | Leaf | 3,55 |  |
|  | ERR2639631 | SAMEA4729065 | Diploid female | Sexually mature | Leaf | 28,3 |  |
|  | ERR2639629 | SAMEA4729063 | Diploid male | Sexually mature | Leaf | 3,97 |  |
|  | ERR2639626 | SAMEA4729060 | Diploid female | Sexually mature | Leaf | 5,33 |  |
|  | ERR2639637 | SAMEA4729071 | Diploid female | Sexually mature | Flowers | 4,51 |  |
|  | ERR2639625 | SAMEA4729059 | Diploid female | Sexually mature | Leaf | 7,15 |  |
